# Redox Brake Regulator RedB and FnrL Function as Yin-Yang Regulators of Anaerobic-Aerobic Metabolism in Rhodobacter capsulatus

**DOI:** 10.1128/spectrum.02354-22

**Published:** 2022-09-15

**Authors:** Nijia Ke, Joseph E. Kumka, Mingxu Fang, Brian Weaver, Judith N. Burstyn, Carl E. Bauer

**Affiliations:** a Molecular and Cellular Biochemistry Department, Indiana University, Bloomington, Indiana, USA; b Department of Chemistry, University of Wisconsin—Madison, Madison, Wisconsin, USA; Griffith University

**Keywords:** FNR ortholog, transcriptomics, redox regulation, global transcription factor binding, photosynthetic bacterium

## Abstract

We recently described a new member of the CRP (cyclic AMP receptor protein)/FNR (fumarate and nitrate reductase regulatory protein) family called RedB, an acronym for redox brake, that functions to limit the production of ATP and NADH. This study shows that the RedB regulon significantly overlaps the FnrL regulon, with 199 genes being either directly or indirectly regulated by both of these global regulatory proteins. Among these 199 coregulated genes, 192 are divergently regulated, indicating that RedB functions as an antagonist of FnrL. Chromatin immunoprecipitation sequencing (ChIP-seq) analysis indicates that RedB and Fnr directly coregulate only 4 out of 199 genes. The primary mechanism for the divergent regulation of target genes thus involves indirect regulation by both RedB and FnrL (156 cases). Additional regulation involves direct binding by RedB and indirect regulation by FnrL (36 cases) or direct binding by FnrL and indirect regulation by RedB (3 cases). Analysis of physiological pathways under direct and indirect control by these global regulators demonstrates that RedB functions primarily to limit energy production, while FnrL functions to enhance energy production. This regulation includes glycolysis, gluconeogenesis, photosynthesis, hydrogen oxidation, electron transport, carbon fixation, lipid biosynthesis, and protein synthesis. Finally, we show that 75% of genomes from diverse species that code for RedB proteins also harbor genes coding for FNR homologs. This cooccurrence indicates that RedB likely has an important role in buffering FNR-mediated energy production in a broad range of species.

**IMPORTANCE** The CRP/FNR family of regulatory proteins constitutes a large collection of related transcription factors, several of which globally regulate cellular energy production. A well-characterized example is FNR (called FnrL in Rhodobacter capsulatus), which is responsible for regulating the expression of numerous genes that promote maximal energy production and growth under anaerobic conditions. In a companion article (N. Ke, J. E. Kumka, M. Fang, B. Weaver, et al., Microbiol Spectr 10:e02353-22, 2022, https://doi.org/10.1128/Spectrum02353-22), we identified a new subgroup of the CRP/FNR family and demonstrated that a member of this new subgroup, called RedB, has a role in limiting cellular energy production. In this study, we show that numerous genes encompassing the RedB regulon significantly overlap genes that are members of the FnrL regulon. Furthermore, 97% of the genes that are members of both the RedB and FnrL regulons are divergently regulated by these two transcription factors. RedB thus functions as a buffer limiting the amount of energy production that is promoted by FnrL.

## INTRODUCTION

Rhodobacter capsulatus is a metabolically versatile photosynthetic bacterium that grows under a wide variety of conditions. Examples include (i) heterotrophic growth using exogenous carbon sources under aerobic or anaerobic conditions, (ii) photosynthetic growth under anaerobic conditions in the presence of infrared light, and (iii) chemoautotrophic growth, with energy and carbon derived from inorganic compounds such as hydrogen and carbon dioxide, respectively (1, 2). Transcriptome analyses have shown that the ability of cells to grow under such disparate conditions requires large-scale changes in cellular physiology (3, 4). For example, comparative transcriptome sequencing (RNA-seq) analysis of R. capsulatus cells grown under dark aerobic (respiratory) versus anaerobic photosynthetic conditions shows that 53% of the genome (1,834 genes) exhibits changes in expression between these two growth conditions (4). Such large-scale changes in gene expression require coordination among numerous global, and specialized, transcription factors to ensure that appropriate genes are expressed to provide competitive growth advantages to the cell.

Several global transcription factors in R. capsulatus that are responsible for controlling aerobic-to-anaerobic changes in gene expression have been well characterized. One of these global regulators is the RegB-RegA two-component system that controls the expression of 591 genes in response to changes in cellular redox (4, 5). In this regulatory system, the sensor kinase RegB responds to changes in cellular redox via a redox-reactive cysteine (6, 7) and by directly interacting with the ubiquinone pool (8, 9). For example, when RegB binds oxidized ubiquinone, its kinase activity is low, but when it binds reduced ubiquinol, its kinase activity is high (8, 9). Another well-characterized global regulator controlling R. capsulatus anaerobic physiology is FnrL, an ortholog of the Escherichia coli fumarate and nitrate reductase regulator protein (FNR) (3, 10). Anaerobically, FNR contains a stable 4Fe-4S cluster that promotes FNR dimerization, DNA binding, and subsequent gene regulation (11, 12). Aerobically the iron-sulfur cluster disassembles, which disrupts FNR’s ability to dimerize and bind DNA (11, 12). Transcriptome analyses of FnrL in the photosynthetic species Rhodobacter sphaeroides and R. capsulatus show that these FNR orthologs function as global regulators of anaerobic physiology by directly and indirectly controlling the expression of 917 and 807 genes, respectively (3, 13). However, although these two *Rhodobacter* species exhibit similar metabolic capabilities, these FnrL orthologs regulate only 171 genes in common (~80% of the genes regulated by FnrL in these two species are uniquely controlled). Furthermore, chromatin immunoprecipitation sequencing (ChIP-seq) analyses also demonstrated that only 9 gene orthologs are directly regulated by FnrL in both *Rhodobacter* species. This number is surprisingly low given that the FnrL proteins in these two species utilize very similar DNA recognition sequences (3, 13). Thus, FnrL orthologs in species with similar anaerobic metabolic challenges and features have dissimilar regulatory roles.

In a companion article, we provide the first genetic and transcriptomic analyses of a member of a previously uncharacterized subclass of the CRP (cyclic AMP receptor protein)/FNR regulatory family termed RedB, an acronym for redox brake (14). RedB was shown to function as a global anaerobic regulator of 451 genes in a manner that limits the production of ATP and NADH (14). RedB also has a significant role in limiting protein synthesis by repressing the synthesis of proteins involved in translation initiation, tRNA synthesis, tRNA charging, and amino acid biosynthesis.

In this study, we directly compare the RedB regulon to the FnrL regulon under photosynthetic anaerobic growth conditions. Our analysis shows that 199 genes are regulated by both of these global regulatory proteins. Strikingly, among the 199 coregulated genes, 192 are divergently regulated by RedB and FnrL, indicating that RedB functions as an antagonist of FnrL.

## RESULTS AND DISCUSSION

### Overview of genes coregulated by FnrL and RedB.

**FIG 1:**
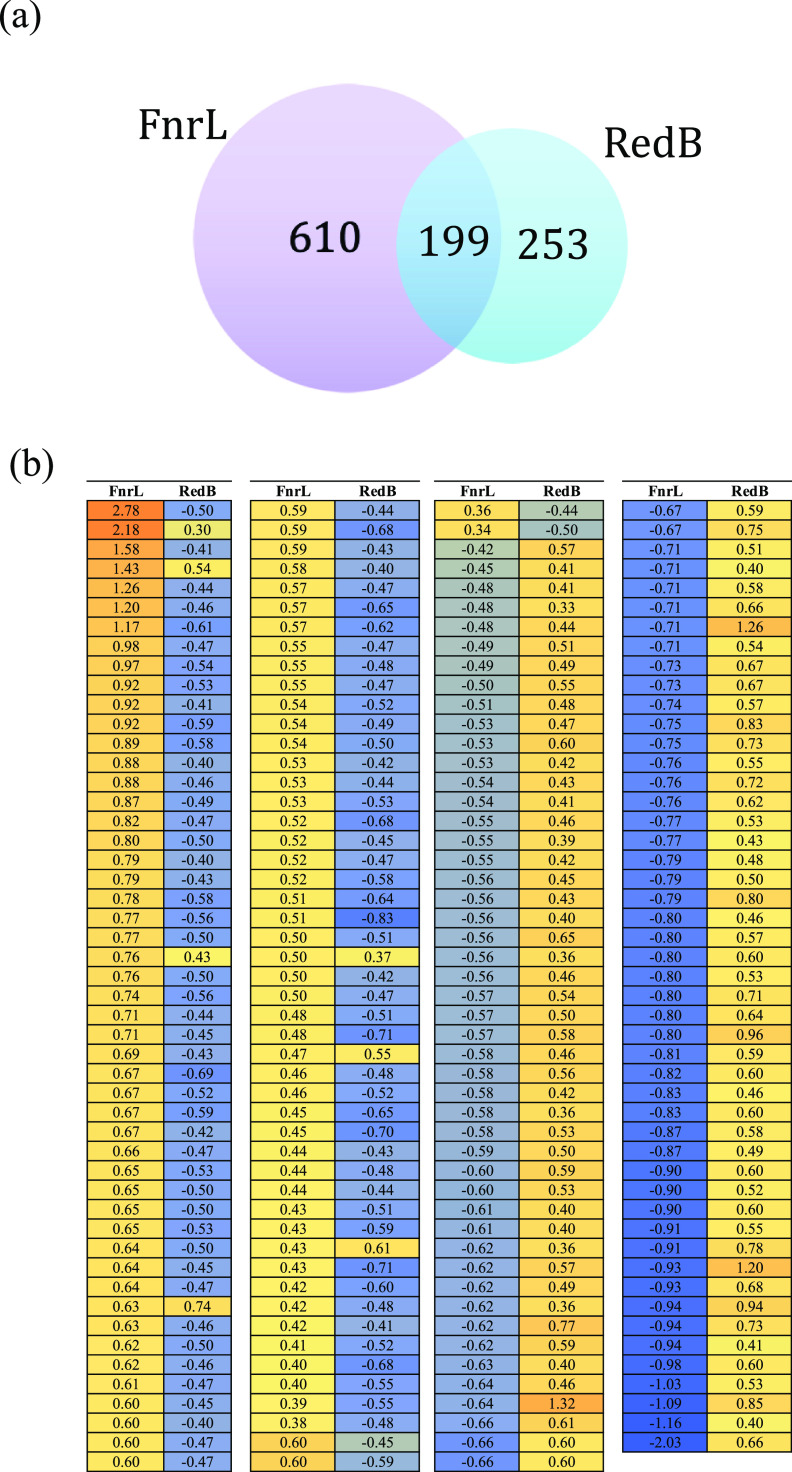
Venn diagram and heat map of genes coregulated by FnrL and RedB. (a) Venn diagram showing that 199 genes are coregulated by FnrL and RedB. (b) Heat map of the same 199 coregulated genes that highlight divergent gene control by FnrL and RedB. Orange-yellow boxes represent genes undergoing activation, while blue boxes represent those undergoing repression. Each line in the FnrL-RedB-paired descending columns represents a different gene. Details of gene names and functions can be found in Table S1 in the supplemental material.

Details of genes individually regulated by RedB and FnrL have been reported elsewhere (3, 14). These studies determined that the RedB deletion strain altered the expression of 451 genes, while the FnrL deletion strain altered the expression of 807 genes, relative to wild-type cells under anaerobic photosynthetic growth conditions. In this study, we focused on comparative analysis of the RedB and FnrL regulons using these previously described RNA-seq and ChIP-seq data sets (3, 14). This analysis demonstrates that 199 genes undergo changes in gene expression in both the FnrL and RedB data sets, which corresponds to 44% of the RedB regulon and 25% of the FnrL regulon (Fig. 1a; see also Table S1 in the supplemental material). Surprisingly, the heat map in Fig. 1b highlights that among these 199 coregulated genes, an extraordinary 96% (192 genes) are regulated in opposing directions by RedB and FnrL (Fig. 1b).

We also investigated the mechanism by which RedB and FnrL undertake divergent control of cellular processes by analyzing previously described ChIP-seq data sets to determine which genes are directly regulated by RedB and FnrL (Table S2). For this analysis, we compared the locations of the binding of RedB and FnrL to the chromosome using previously called ChIP-seq peaks that exhibited a false discovery rate (FDR) cutoff of 5% (corresponding to an unadjusted *P* value of <1E−5) (3, 14). This analysis shows that among the 199 genes that are coregulated by RedB and FnrL, 40 genes are directly regulated by RedB based on the presence of a ChIP-seq peak near genes that exhibit expression changes when *redB* is deleted (Table S2-1). The majority of these (32 genes) are repressed by RedB, with all but one of them being divergently regulated by FnrL, mostly via an indirect mechanism. Regarding FnrL, among the 199 coregulated genes, 7 are directly regulated by FnrL, with all of them being activated by FnrL. Again, about one-half of these genes (three genes) are indirectly regulated by RedB (Table S2-2). Finally, only four genes are directly coregulated by both RedB and FnrL (Table S2-3).

### Downstream transcription control mechanisms.

**FIG 2:**
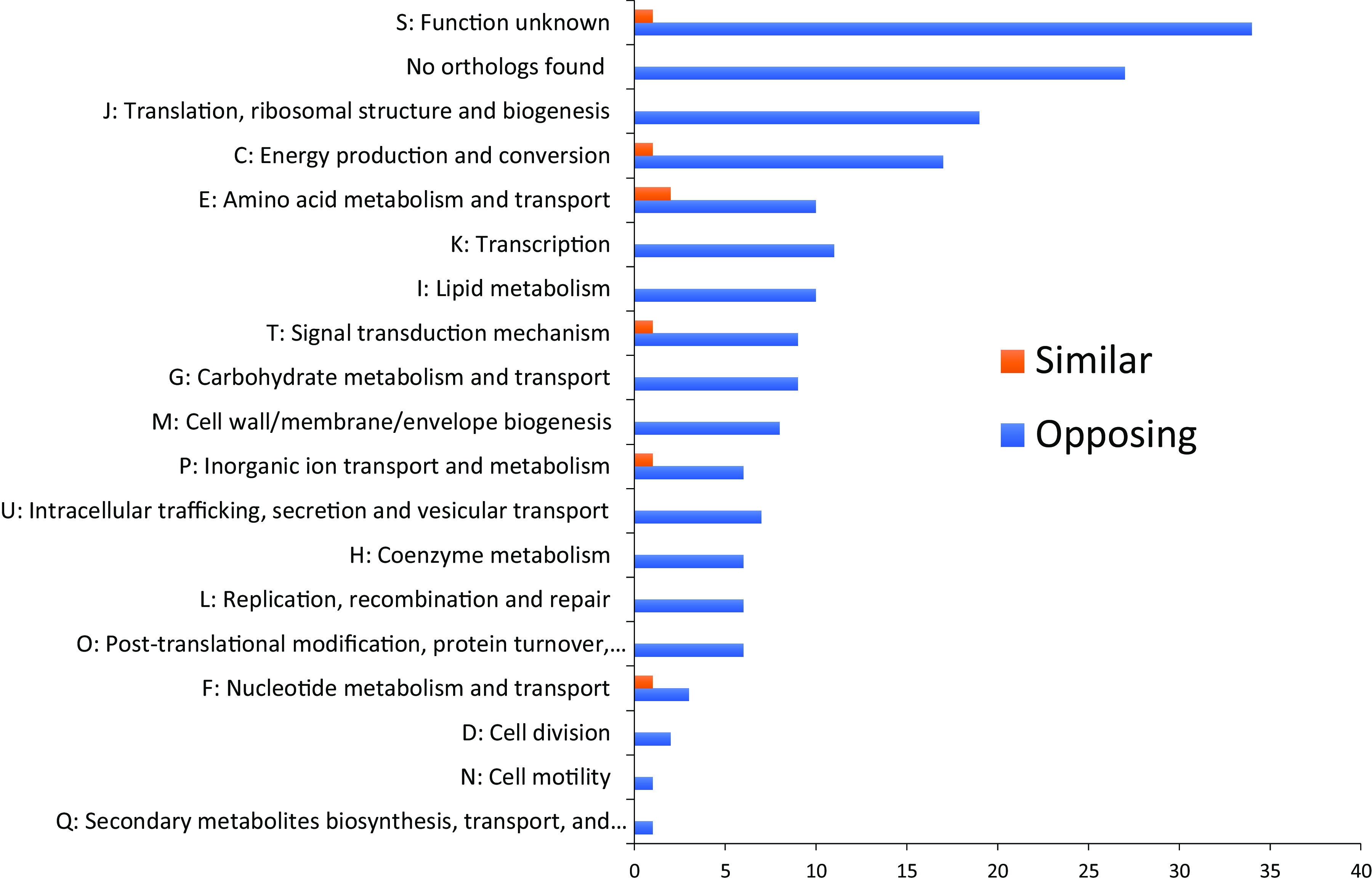
COG assignment of overlapping genes. Shown is a bar chart of the number of overlapping genes regulated by both FnrL and RedB classified into different COGs, with the genes convergently regulated represented by orange bars and the ones divergently regulated represented by blue bars.

We sought to deduce the mechanism by which RedB and FnrL undertake divergent coregulation of so many genes, given that only four genes are directly coregulated by both of these global regulators. For this analysis, we binned each of the 199 coregulated genes into different cognate Clusters of Orthologous Groups (COGs) (Table S3), with the bar graph in Fig. 2 showing convergently and divergently regulated genes in different COG groups. As indicated in Fig. 2, COGs that draw attention to divergent regulation are COG K (transcription) and COG T (signal transduction mechanism). A detailed examination of these regulatory genes shows that RedB and FnrL divergently affect the expression of 11 transcription factors, 2 histidine sensor kinases, 2 OmpR family member response regulators, and several diguanylate cyclases/phosphodiesterases (Table S2-5). Notable is the observation that RedB directly represses, while FnrL indirectly activates, the expression of *rpoA*, which codes for the alpha subunit of DNA-directed RNA polymerase. The amino-terminal region of the alpha subunit initiates the assembly of the RNA polymerase holoenzyme (15), while the carboxyl region interacts with class I transcription factors, including members of the CRP family such as RedB and FnrL (16–19). Thus, the divergent regulation of *rpoA* may exert significant global effects on gene expression. Another example is *carD*, which is directly repressed by RedB and directly activated by FnrL. In nonenteric bacteria, CarD is thought to interact with the RNA polymerase holoenzyme as a pseudo subunit. Specifically, evidence suggests that CarD interacts with both the beta subunit of RNA polymerase and the upstream edge of the transcription bubble to function as a wedge that prevents transcription bubble collapse (20–23). Similar to *rpoA*, it is plausible that the divergent regulation of CarD expression could affect the transcription of a plethora of downstream genes.

### Divergent roles of RedB and FnrL as controllers of energy production.

Inspection of Fig. 2 also shows that one of the larger groups undergoing divergent control by RedB and FnrL is COG C, “energy production and conversion.” This suggests that RedB and FnrL counterbalance each other in energy metabolism. An analysis of this divergent control is described below.

### (i) Glycolysis versus gluconeogenesis.

**FIG 3:**
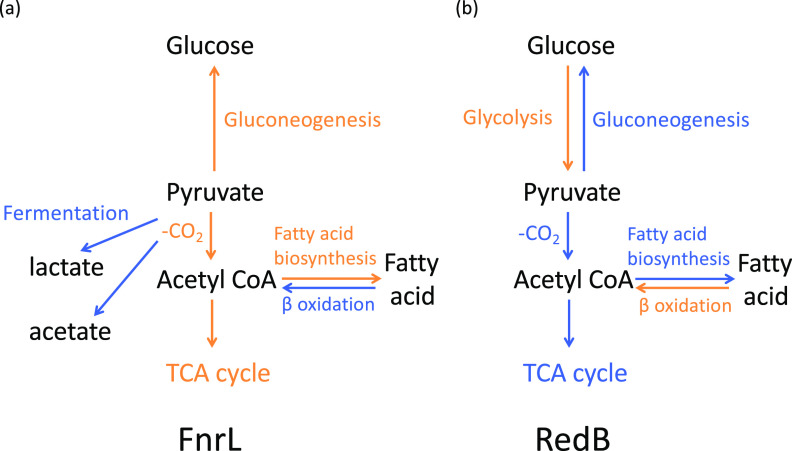
Summary of the regulation effects of FnrL and RedB on central metabolic pathways. (a) FnrL activates pyruvate decarboxylation to acetyl-CoA and activates the synthesis of enzymes involved in the TCA cycle to produce more reducing equivalents in the form of NADH and FADH_2_. Accordingly, FnrL inhibits pyruvate fermentation to lactate or acetate, avoiding this inefficient metabolic process in terms of energy production. In the anabolic direction, FnrL promotes gluconeogenesis and fatty acid biosynthesis, whereas in the catabolic direction, FnrL inhibits β-oxidation. (b) In contrast to FnrL, RedB inhibits pyruvate decarboxylation to acetyl-CoA as well as the synthesis of enzymes involved in the TCA cycle to prevent reducing equivalents from being generated for further energy production. Not only are fatty acid biosynthesis and gluconeogenesis inhibited by RedB, but also the catabolic pathways in the opposite direction, namely, β-oxidation and glycolysis, are activated by RedB to break down macromolecules, including fatty acids and carbohydrates. Pathways repressed by FnrL or RedB are highlighted in blue, whereas pathways activated by FnrL or RedB are highlighted in orange.

One overarching theme is that RedB and FnrL divergently control the flow of carbon through central metabolism (Fig. 3). For example, with respect to feeding sugars into glycolysis, R. capsulatus codes for several ABC transport systems (RCC02544 and RCC03235) thought to be involved in the transport of simple sugars and *N*-acetyl-d-glucosamine. The *nagA* gene also encodes the enzyme *N*-acetylglucosamine-6-phosphate deacetylase, which degrades *N*-acetylglucosamine-6-phosphate into fructose-6-phosphate. In line with evidence that RedB promotes glycolysis while FnrL promotes gluconeogenesis, the RNA-seq and Chip-seq data sets show that RedB indirectly activates the expression of these ABC transporters and *nagA*. Conversely, FnrL indirectly represses the expression of each of these genes (Table S3-1). Additionally, enzymes of the Leloir pathway (also referred to as the galactose degradation pathway) are involved in the conversion of d-galactose to the more metabolically versatile d-glucose-6-phosphate, which also feeds into glycolysis. Similar to *N*-acetylglucosamine transport and degradation, RedB indirectly upregulates while FnrL indirectly downregulates the expression of *galM*, which codes for galactose mutarotase. This enzyme catalyzes the first step in the Leloir pathway (Table S3-1).

Two enzymes involved in key steps of glycolysis are 6-phosphofructokinase (*pfkB*) and pyruvate kinase (*pykA2*), both of which are significantly indirectly activated by RedB (Table S3-1). These two enzymes catalyze the irreversible phosphorylation of fructose-6-phosphate into fructose 1,6-bisphosphate and the irreversible dephosphorylation of PEP (phosphoenolpyruvate) into pyruvate, respectively. Consequently, RedB-mediated expression of these genes would favor an increase in glycolysis. Concurrently, RedB represses the expression of two different enzymes that promote the reverse flow of carbon (gluconeogenesis) at the same steps in the pathway. Specifically, RedB indirectly represses fructose 1,6-bisphosphatase, encoded by *fbp*, and directly represses phosphoenolpyruvate carboxykinase, encoded by *pckA* (Tables S2-1 and S3-1). In addition, RedB indirectly represses the synthesis of malate dehydrogenase (*maeB2*), which catalyzes the irreversible initiation step in gluconeogenesis (Table S3-1). Thus, RedB appears to stimulate glycolysis while concurrently repressing the reverse-flowing process of gluconeogenesis.

In contrast to RedB, FnrL has an opposing role in gluconeogenesis. For example, FnrL indirectly activates phosphoenolpyruvate carboxykinase (*pckA*) and malate dehydrogenase (*maeB2*), which would stimulate gluconeogenesis (Table S3-1). Interestingly, type I glyceraldehyde-3-phosphate dehydrogenase (*gap3*), shared by the glycolysis and gluconeogenesis pathways, is directly repressed by RedB and indirectly activated by FnrL (Tables S2-1 and S3-1). Taken together, these results demonstrate that RedB favors glycolysis, while FnrL favors reverse carbon flow via gluconeogenesis.

Further down the central metabolic pathway, FnrL indirectly activates the expression of the alpha subunit of pyruvate dehydrogenase (*pdhA*), while RedB indirectly represses the expression of the beta subunit of pyruvate dehydrogenase (*pdhB*) and indirectly suppresses the expression of dihydrolipoyl dehydrogenase (*lpdA1*) (Table S3-1). All three are components of pyruvate dehydrogenase that catalyze the decarboxylation of pyruvate to acetyl-CoA. This suggests that FnrL activates pyruvate decarboxylation to acetyl-CoA either to produce more energy through the tricarboxylic acid (TCA) cycle or to allow increasing fatty acid synthesis, while RedB inhibits pyruvate decarboxylation to acetyl-CoA to reduce energy generation.

### (ii) TCA cycle.

Concerning the TCA cycle, FnrL indirectly stimulates the expression of numerous enzymes in this cycle, such as isocitrate dehydrogenase (*icd*), 2-oxoglutarate (α-ketoglutarate) dehydrogenase (*sucA*), succinyl-CoA synthetase subunit β (*sucC*), succinate dehydrogenase (*sdhB*), dihydrolipoyllysine succinyltransferase (*sucB*), and fumarate hydratase (*fumC*) (Table S2-1). In contrast, RedB indirectly downregulates the expression of *sucA*, *sucB*, *sdhB*, and *fumC* (Table S3-1). The flow of carbon through this cycle leads to the production of significant reducing equivalents in the form of NADH and reduced flavin adenine dinucleotide (FADH_2_) as well as ATP. Thus, it appears that FnrL favors the production of large amounts of energy by activating the expression of TCA cycle enzymes, while RedB appears to reduce energy production by repressing the synthesis of several TCA cycle enzymes (Fig. 3).

Finally, FnrL indirectly activates *dctQ2*, which encodes a subunit of a high-affinity ABC transporter that feeds several intermediates into the TCA cycle, including malate, fumarate, and succinate (24). Conversely, RedB indirectly represses *dctQ2* expression (Table S3-1).

### (iii) Nicotinamide metabolism.

FnrL indirectly downregulates the expression of *ppnK*, which encodes the enzyme NAD kinase that converts NAD^+^ into NADP^+^. Conversely, RedB not only indirectly upregulates the expression of *ppnK* but also indirectly downregulates the expression of *pntA* and *phoA*, encoding NADP transhydrogenase and alkaline phosphatase, which together catalyze the reverse reaction, namely, the conversion of NADP^+^ to NAD^+^ (Table S3-1). These converse expression patterns show that FnrL favors the presence of NAD^+^ over that of NADP^+^, while RedB favors the opposite. Interestingly, NAD^+^ is generally more involved in energy production for central metabolism, while NADP^+^ is more often used in anabolic reactions.

### (iv) Photosynthesis, fatty acid biosynthesis, and degradation.

**FIG 4:**
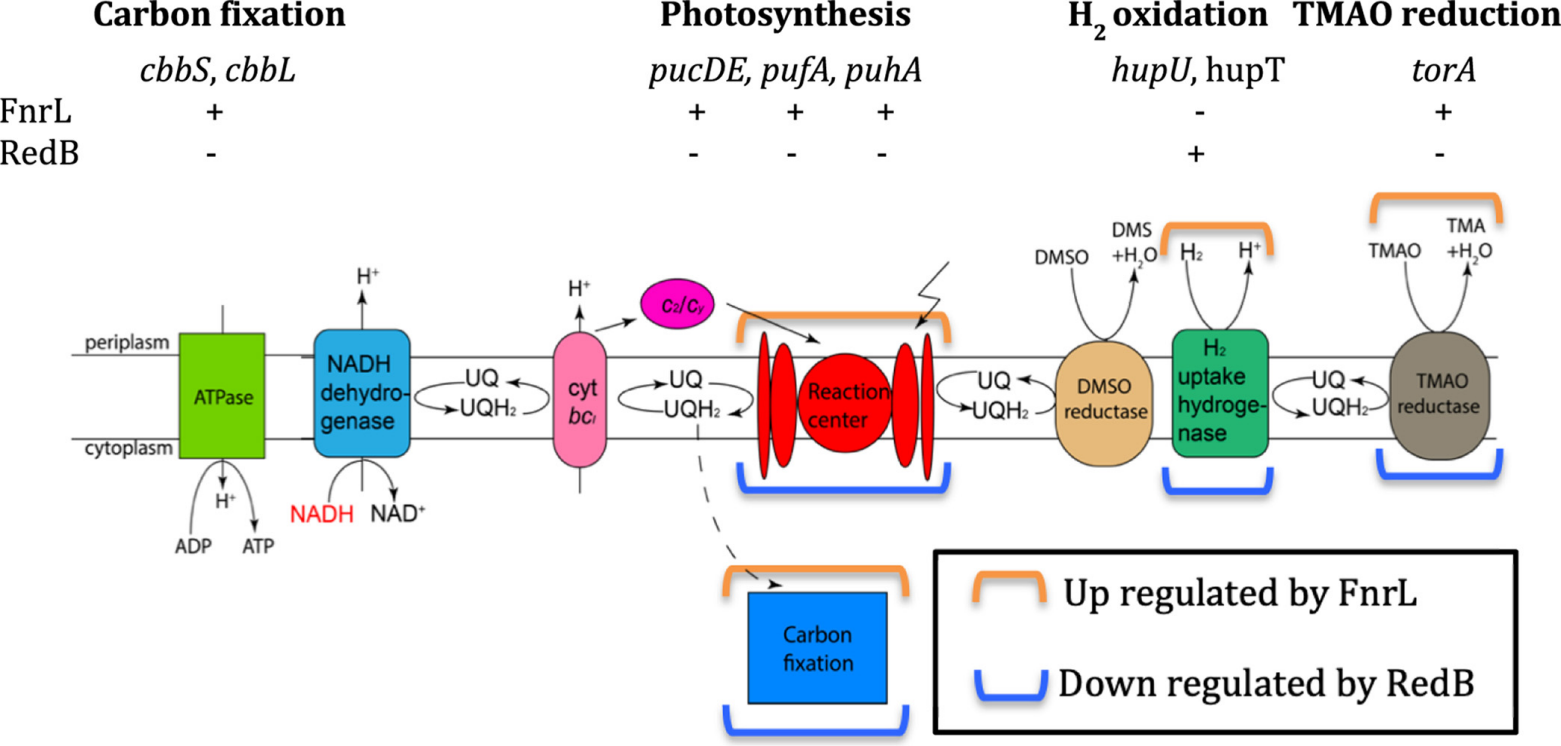
Schematic of R. capsulatus anaerobic cellular activities. The activation or repression of genes is indicated by + or −, respectively. For simplicity, only coregulated genes of FnrL and RedB are shown. The corresponding directions of regulation of each cellular activity by FnrL and RedB are marked with brackets according to the key in the box. DMSO, dimethyl sulfoxide reductase; TMAO, trimethylamine N-oxide reductase.

In a previous study, we demonstrated that FnrL activates the expression of AerR, which is a B_12_-dependent photoactivated regulator of photosynthesis gene expression (3). AerR functions by converting a well-characterized repressor of photosynthesis, CrtJ, into an activator of photosynthesis gene expression (25–27). Thus, it is not surprising that many photosynthesis genes, such as *pucDE*, *pufAB*, *puhA*, and *bchE*, are all indirectly activated by FnrL (Fig. 4 and Table S3-3) (3). In the context of divergent regulation, several photosystem structural genes (*pucDE*, *bchE*, and *crtA*) are directly repressed by RedB (Table S2-1). This indicates that FnrL activates the synthesis of the photosystem, whereas RedB inhibits its synthesis.

The R. capsulatus photosystem is housed in an internal “intracytoplasmic membrane” (ICM) complex (28). Thus, the synthesis of the photosystem is concurrent with the increased synthesis of fatty acids/lipids. Inspection of genes coding for enzymes involved in fatty acid biosynthesis shows that a key enzyme, acetyl-CoA carboxylase carboxyl transferase subunit β (*accD*), is indirectly activated by FnrL and indirectly repressed by RedB (Table S3-1). This indicates that FnrL promotes the initiation of fatty acid biosynthesis, while RedB inhibits it. In addition, FnrL also indirectly upregulates the expression of 3-oxoacyl-ACP (acyl carrier protein) synthase III (*fabH1*), which catalyzes a further irreversible step in the initiation of fatty acid biosynthesis. During the process of fatty acid elongation and maturation, another enzyme, enoyl-acyl carrier protein reductase (*fabI1*), is also directly activated by FnrL and indirectly inhibited by RedB (Tables S2-2 and S3-1). The latter enzyme might regulate fatty acid elongation when there is a surplus of reducing equivalents, as this step harnesses NADH. However, another enoyl-acyl carrier protein reductase (*fabI*) that catalyzes the same reaction is indirectly repressed by FnrL but indirectly activated by RedB, although the fold changes are not as large (Table S3-1). This second enzyme perhaps functions as a mitigator when there is not enough NADH generated in R. capsulatus.

The catabolism of fatty acids to generate acetyl-CoA is also divergently regulated. For example, in the first step of dehydrogenation in the β-oxidation cycle, the expression of acyl-CoA dehydrogenase (*rcc00405*), which is unique to this pathway, is indirectly downregulated by FnrL and indirectly upregulated by RedB (Table S3-1). This suggests that FnrL inhibits fatty acid degradation, whereas RedB activates this process. In addition, the expression of fatty acid oxidation complex subunit α (*fadB*), an enzyme that catalyzes the second and third steps in the β-oxidation cycle, is also indirectly downregulated by FnrL (Table S3-1). Similarly, FnrL indirectly represses the expression of 3-hydroxy-2-methylbutyryl-CoA dehydrogenase (*hadH*), which is also involved in the third step of dehydrogenation in the β-oxidation cycle (Table S3-1).

Overall, it appears that FnrL promotes the initiation of fatty acid biosynthesis and concurrently inhibits fatty acid degradation, whereas RedB restrains the initiation of fatty acid biosynthesis while activating fatty acid degradation. These effects are consistent with the divergent roles of FnrL and RedB in the synthesis of the photosystem, which is housed in the ICM.

### (v) Hydrogen oxidation.

The energy-producing uptake hydrogenase converts H_2_ to H^+^ with electrons shuttled to ubiquinone during its conversion to ubiquinol (Fig. 4). The expression of hydrogenase structural genes is regulated by the HupT/HupR two-component system, in which HupT is a histidine kinase and HupR is a cognate response regulator (29, 30). In the absence of H_2_, two other proteins, HupU and HupV, are involved in repressing hydrogenase structural genes in concert with *hupT* (29). When H_2_ is present, nonphosphorylated HupR directly activates the *hupSLC* gene cluster, which codes for hydrogenase (30). RNA-seq and ChIP-seq results show that the expression levels of *hupU* and *hupT* are both indirectly downregulated by FnrL and, conversely, indirectly upregulated by RedB (Table S3-3). This indicates that energy production as derived through H_2_ oxidation is activated by FnrL and repressed by RedB.

In addition to the membrane-bound uptake hydrogenase, FnrL indirectly activates, and RedB indirectly represses, the expression of *hoxH*, which codes for a cytosolic bidirectional NAD-reducing hydrogenase (Table S3-3). This enzyme couples the oxidation and reduction of NAD with the alternating oxidation/reduction of hydrogen.

### (vi) Electron transport.

**FIG 5:**
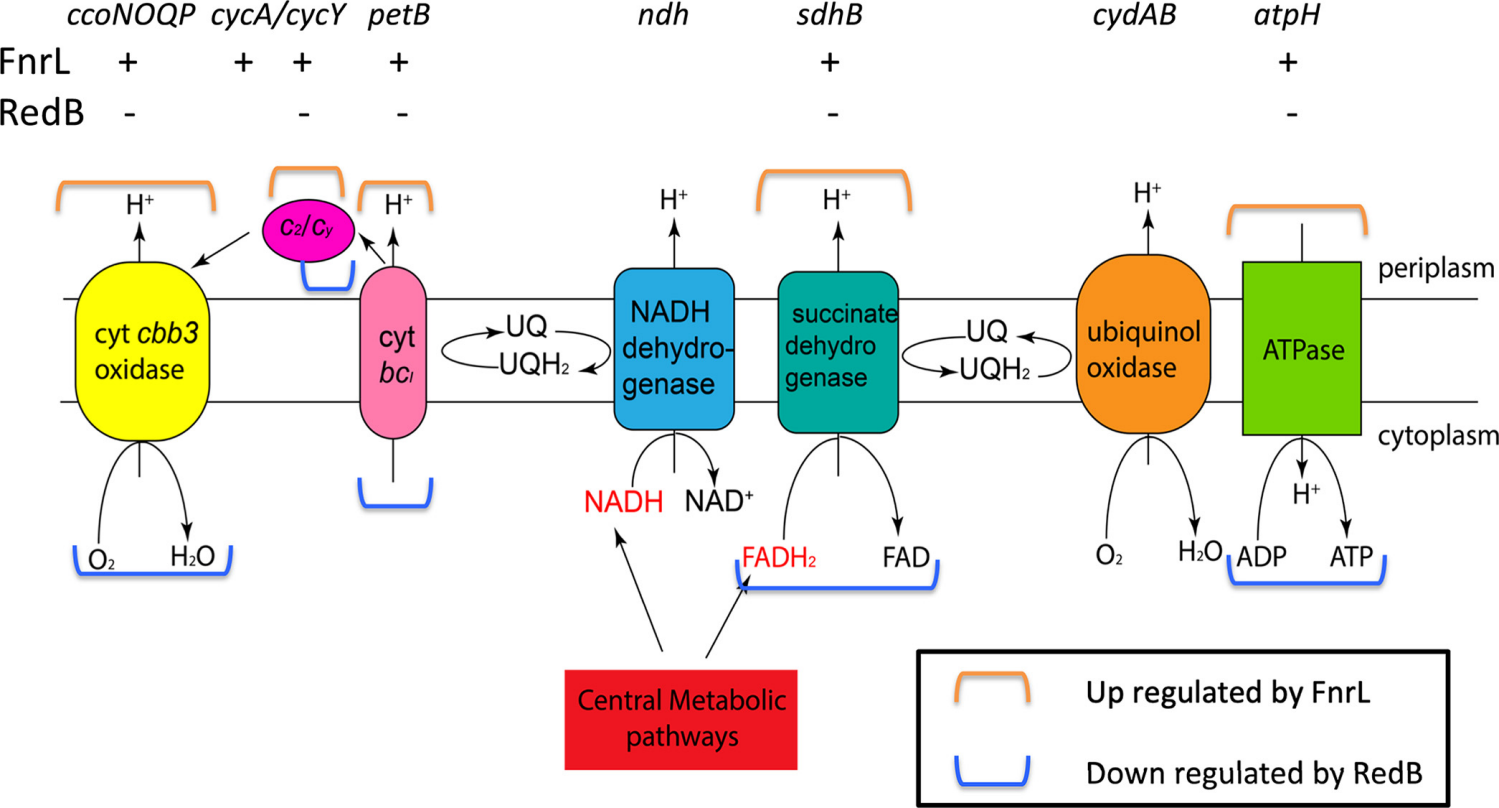
Schematic of the R. capsulatus electron transport chain. The activation or repression of genes is indicated by + or −, respectively. For the gene clusters *sdhABCD* and *atpABCDEFGHIX*, only coregulated genes of FnrL and RedB are shown for simplicity. The corresponding directions of regulation of the synthesis of each complex by FnrL and RedB are marked with brackets according to the key in the box.

Reducing equivalents stored in the form of NADH and FADH_2_ can be shuttled to ubiquinone (UQ) to form reduced ubiquinol (UQH_2_) via membrane-bound NADH dehydrogenase and/or succinate dehydrogenase (Fig. 5) (31). As noted above, photosynthesis and hydrogenase also generate reduced ubiquinol. Reducing equivalents in UQH_2_ can be directly utilized by ubiquinol oxidase to reduce O_2_ to H_2_O. Alternatively, electrons in UQH_2_ can be shuttled to cytochrome *cbb*_3_ oxidase via cytochrome *c*_2_ or back to the photosystem via cytochrome *c*_y_ after reoxidation by cytochrome *bc*_1_ complex Q (32). Protons are pumped from the cytosol into the inner membrane at several steps in this electron transport process, with the resulting proton gradient being used by ATPase to generate ATP (Fig. 5).

As shown in Fig. 5 and Table S3-2, there are several components in the electron transfer chain described above that are activated by FnrL but repressed by RedB. Notably, genes coding for subunits of the succinate dehydrogenase complex (*sdhB*, *sdhC*, and *sdhD*) are indirectly activated by FnrL. At the same time, *sdhB* is also indirectly repressed by RedB. This indicates that FnrL facilitates the transfer of electrons from FADH_2_ (33) to the electron transport chain to produce more energy, while RedB inhibits this activity.

Further along the electron transport chain, the expression of subunit *b* of the cytochrome *bc*_1_ complex (ubiquinol-cytochrome *c* reductase), encoded by *petB*, is also boosted by FnrL and reduced by RedB (Table S2-2). Furthermore, the expression of other electron carriers, such as cytochrome *c*_y_, encoded by *cycY*, and cytochrome *c*_2_, encoded by *cycA1*, is indirectly activated by FnrL while also being indirectly repressed by RedB (Table S3-2). Finally, FnrL indirectly upregulates the expression of *ccoN*, coding for subunit I of cytochrome *cbb*_3_ oxidase, while RedB indirectly downregulates the expression of subunit II (*ccoO*) and subunit IV (*ccoQ*) (Table S3-2).

In the presence of Trimethylamine N-oxide (TMAO) and dimethyl sulfoxide (DMSO), TMAO-DMSO reductase can serve as an additional terminal electron acceptor to accept electrons from ubiquinol (Fig. 4) (34). It is known that FnrL indirectly activates both *torC* and *torA*, which code for TMAO reductase *c*-type cytochrome and TMAO reductase, respectively, promoting TMAO reduction to keep the redox potential (3). In contrast, RedB indirectly represses *torA*, which would inhibit TMAO reduction (Table S3-2). It is also known that the activation of both the *torCA* and *dorCDA* operons, encoding TMAO and DMSO reductases, is regulated by the DorS/DorR two-component system (35, 36). We notice that the expression of *dorS*, which encodes the DMSO-TMAO sensor hybrid histidine kinase, is directly upregulated by both RedB and FnrL although at different levels (Tables S2-3 and S3-2). Specifically, FnrL directly upregulates the expression of the *dorS* gene by 4.5-fold, whereas RedB directly upregulates *dorS* by only 1.23-fold. This gene is one of only four genes that are directly regulated by both RedB and FnrL.

Regarding ATP synthase encoded by the *atpABCDEFGHIX* gene cluster (37), the expression of the ATP synthase F_1_ delta subunit (*atpH*) is coregulated by both FnrL and RedB, with *atpH* being indirectly activated by FnrL but also directly repressed by RedB (Fig. 5 and Tables S2-1 and S3-2). In line with this observation, RedB also inhibits the expression of *atpA*, *atpB*, and *atpG*, which code for ATP synthase F_1_ subunit alpha, ATP synthase F_o_ subunit A, and ATP synthase F_1_ subunit gamma, respectively. FnrL also indirectly enhances the expression of *atpI*, which codes for ATP synthase F_o_ subunit I. However, different from what would be expected, FnrL indirectly represses the expression of *atpC*, which encodes ATP synthase F_1_ subunit epsilon. It is unknown why this single gene is regulated in the opposite direction.

Overall, it is clear that RedB represses several components in the electron transport chain responsible for generating a proton gradient and the synthesis of ATPase that utilizes this proton gradient to synthesize ATP. This is in stark contrast to FnrL, which promotes the expression of electron transport chain components leading to significant ATP synthesis.

### (vii) Carbon fixation.

Carbon fixation provides cells with fixed carbon and also functions as an electron sink to help balance cellular redox (38). Inspection of genes coding for enzymes in the Calvin-Benson-Bassham (CBB) cycle shows that *cbbL* and *cbbS*, coding for the large and small subunits of ribulose bisphosphate carboxylase (RuBisCo), are indirectly upregulated by FnrL (Fig. 4 and Table S3-3). Conversely, RedB indirectly suppresses the expression of these same genes and the expression of several additional genes (*cbbM*, *cbbP*, and *fbp*) in the CBB cycle. This result suggests that FnrL promotes carbon fixation, while RedB inhibits carbon fixation.

### Divergent roles of RedB and FnrL in controlling protein synthesis and folding. (i) Protein synthesis.

Regarding protein synthesis, evidence indicates that RedB represses, and FnrL stimulates, this essential function. Specifically, FnrL indirectly activates 13 genes coding for the 30S and 50S ribosomal proteins; *infA*, which encodes the translation initiation factor IF-1; and *efp*, which encodes translation elongation factor P. All of these genes are repressed by RegB (Tables S2-1 and S3-4). Notably, RedB directly represses the expression of *rpmI* and *rpmG*, which code for the 50S ribosomal proteins L35 and L33, respectively (Table S2-1). Conversely, FnrL directly activates the expression of L35 and indirectly activates the expression of L33 (Table S2-2). Additionally, RedB directly represses the expression of *rpsM* and *rpsA*, while FnrL indirectly activates the expression of these genes (Tables S2-1 and S3-4). *rpsM* codes for 30S ribosomal subunit S13, which binds fMet-tRNA and is thus involved in the initiation of translation (39). S13 also affects the strength of small and large ribosomal subunit interactions (39). *rpsA* codes for the small subunit protein S1, which is the largest protein in the 30S subunit. Like S13, S1 also participates in translation initiation, as it is required for the assembly of the 30S initiation complex at Shine-Dalgarno ribosome binding sites (40–43). Indeed, S1 is thought to have a critical role in the initiation of the translation of most mRNAs in a cell (43). Consequently, the divergent activation and repression of S1 likely have significant consequences for a cell’s ability to undertake protein synthesis.

### (ii) Amino acid biosynthesis and tRNA charging.

Regarding amino acid biosynthesis and tRNA charging, FnrL indirectly activates the expression of 18 genes coding for enzymes involved in amino acid biosynthesis and 5 genes involved in tRNA charging. All but four of these genes are repressed by RedB. For rRNA processing, FnrL indirectly downregulates the expression of tetrapyrrole methylase (*rcc00464*), tRNA (uracil-5)-methyltransferase (*rcc00191*), and TrmH family RNA methyltransferase (*rcc02784*), which are involved in the methylation of cytidine in 16S rRNA, uracil in 23S rRNA, and guanosine in 23S rRNA, respectively (Table S3-4) (44). In contrast, RedB indirectly upregulates the expression of these three rRNA processing genes.

### (iii) Protein folding and secretion.

FnrL indirectly activates, and RedB indirectly represses, the expression of *tig*, which codes for a ribosome-associated chaperone trigger factor that prevents growing peptides from misfolding during translation (Table S3-4). FnrL also indirectly activates, and RedB directly represses, the expression of the molecular chaperone DnaK that assists protein folding (45). Interestingly, trigger factor and DnaK cooperatively fold nascent proteins (46–48), with neither being essential; however, the loss of both trigger factor and DnaK leads to the loss of growth at temperatures above 30°C (46, 47). Similarly, the expression of secretory genes coding for the preprotein translocase subunits SecE, SecY, and SecA is indirectly activated by FnrL. Conversely, SecE and SecA are indirectly repressed, and SecY is directly repressed, by RedB (Table S3-4).

Taken together, it is clear that FnrL promotes protein synthesis and protein folding, while RedB functions to inhibit these essential cellular processes.

### Other Coregulated processes.

There are additional divergently regulated genes that are not involved in energy production or protein synthesis. For example, six lipoproteins and five transport proteins (many of which are involved in metal transport) undergo direct and indirect divergent control (Table S3-6). Additionally, *pyrF* is one of four genes divergently directly regulated by both FnrL and RedB (Table S2-3). This gene codes for the orotidine-5′-phosphate decarboxylase that catalyzes the last indispensable step in the *de novo* biosynthesis of pyrimidines (49). Since FnrL directly upregulates but RedB directly downregulates the expression of *pyrF*, it appears that FnrL promotes the synthesis of nucleic acid, whereas RedB represses this process.

Finally, there are 62 additional genes categorized in Fig. 2 as “function unknown” and “no orthologs found,” 61 of which undergo divergent regulation by FnrL and RedB (Tables S2-1 and S3-6). Without functions, it is impossible to ascertain their roles in cellular events such as metabolism, catabolism, or replication. However, it is striking how many genes in this category are divergently regulated. A good example is *rcc00901*, which encodes a hypothetical protein that is directly activated by FnrL and also directly repressed by RedB (Table S2-3). FnrL upregulates *rcc00901* 6.87-fold, the highest fold change among the 199 genes coregulated by FnrL and RedB. Its function in the cell will have to await further analysis.

### Conclusion.

FNR, and its ortholog FnrL, is a well-known global regulator that controls anaerobic metabolism in a wide variety of bacterial species (3, 10, 13). Genes activated by R. capsulatus FnrL promote several anabolic pathways such as fatty acid biosynthesis and gluconeogenesis (Fig. 3). FnrL also activates gene expression responsible for generating large amounts of energy from the TCA cycle and energy from anoxygenic photosynthesis (3). On the other hand, RedB activates catabolic pathways to break down macromolecules while also inhibiting the above-described pathways of energy generation that are favored by FnrL (14). Thus, FnrL and RedB appear to function as reciprocal buffers that coordinately control energy production from a wide variety of metabolic pathways and cellular activities.

The divergent regulatory functions of R. capsulatus RedB raise the question of whether this regulatory system is at play in other bacteria. As a first attempt to address this question, we sought to establish whether there is a cooccurrence of FNR and RedB proteins in other bacterial genomes. The sequence similarity network described in the companion article (14) that defined RedB as an undefined Fnr-like subcluster also generated a curated list of proteins in the FNR, FnrL, and RedB families. We cross-referenced these curated lists to analyze whether the genomes of 104 other species that contain RedB orthologs also contain FNR and FnrL orthologs. From this analysis, we observed that 75% of the genomes that code for RedB proteins also harbor genes coding for FNR or FnrL proteins (Table S4). This observation supports a working hypothesis that RedB and FNR transcription factors act as yin-yang regulators of anaerobic metabolism in a broad range of species. This hypothesis can be tested by undertaking further experimental analyses of RedB and FNR orthologs in these other organisms.

As discussed above, FNR and its orthologs utilize the assembly and oxygen-mediated disassembly of an iron-sulfur cluster to respond to the presence or absence of dioxygen (11, 12, 50, 51). RedB and members of its subgroup, which are mechanistically unexplored, contain a conserved Cys, but it is not in the correct position, nor is it present in requisite numbers, to harbor an iron-sulfur cluster. Future biochemical studies on redox sensing by RedB will be required to ascertain how this unique member of the Fnr class of transcription factors senses changes in cellular redox.

What is the mechanism by which these two related transcription factors reciprocally regulate energy production? Among the 199 genes regulated by both FnrL and RedB, only 4 appear to be directly regulated by both (Table S2-3). This regulation thus relies primarily upon indirect control or a combination of direct control by one regulator and indirect control by the other. The indirect global regulation of gene expression by RedB might be achieved by directly repressing the expression of the alpha subunit of DNA-directed RNA polymerase and CarD, which has a role in stabilizing transcription initiation (20). A similar indirect effect may be achieved via the RedB-mediated repression of the ribosomal protein subunits S13 and S1, which have important roles in translation initiation (39–43). Thus, the control of just a few transcription factors and key ribosomal proteins may have rather profound global effects on protein synthesis. Clearly, additional studies on the mechanism of RedB regulatory control are warranted, as this newly characterized member of the CRP/FNR family appears to have an important role in balancing the amount of cellular energy production.

## MATERIALS AND METHODS

### RNA-seq and ChIP-seq analyses.

The FnrL and RedB RNA-seq and ChIP-seq data sets used in this study were previously deposited and reported by Kumka and Bauer (3) and Ke et al. (14), respectively.

### Calculation of RedB:FNR cooccurrence.

To establish the cooccurrence of RedB and FNR proteins, we used a previously developed sequence similarity network comprised of proteins within the CRP/FNR superfamily (14). The 50% representative node network provided established clusters of RedB, FNR, and FnrL proteins that we used for our analysis. In the RedB cluster, there are 383 nodes that represent a total of 1,240 protein sequences. We chose 104 RedB nodes that were evenly sampled across the RedB cluster, randomly selected a single sequence within each node, and generated a list of organisms that encoded these proteins. By querying the FNR clusters using our list of organisms, we identified the FNR proteins that were encoded by organisms that contained a RedB homolog. Table S4 in the supplemental material lists UniProt accession numbers for all 104 RedB homologs, the corresponding organisms, and the accession numbers for all FNR proteins encoded by those organisms.

## References

[1] Imhoff JF. 1995. Taxonomy and physiology of phototrophic purple bacteria and green sulfur bacteria, p 1–15. *In* Blankenship RE, Madigan MT, Bauer CE (ed), Anoxygenic photosynthetic bacteria. Advances in photosynthesis and respiration, vol 2. Kluwer Academic Publishers, Dordrecht, The Netherlands.

[2] Tichi MA, Tabita FR. 2001. Interactive control of *Rhodobacter capsulatus* redox-balancing systems during phototrophic metabolism. J Bacteriol 183:6344–6354. doi:10.1128/JB.183.21.6344-6354.2001. 11591679 PMC100130

[3] Kumka JE, Bauer CE. 2015. Analysis of the FnrL regulon in *Rhodobacter capsulatus* reveals limited regulon overlap with orthologues from *Rhodobacter sphaeroides* and *Escherichia coli*. BMC Genomics 16:895. doi:10.1186/s12864-015-2162-4. 26537891 PMC4634722

[4] Kumka JE, Schindel H, Fang M, Zappa S, Bauer CE. 2017. Transcriptomic analysis of aerobic respiratory and anaerobic photosynthetic states in *Rhodobacter capsulatus* and their modulation by global redox regulators RegA, FnrL and CrtJ. Microb Genom 3:e000125. doi:10.1099/mgen.0.000125. 29114403 PMC5643017

[5] Schindel HS, Bauer CE. 2016. The RegA regulon exhibits variability in response to altered growth conditions and differs markedly between *Rhodobacter* species. Microb Genom 2:e000081. doi:10.1099/mgen.0.000081. 28348828 PMC5359404

[6] Swem LR, Kraft BJ, Swem DL, Setterdahl AT, Masuda S, Knaff DB, Zaleski JM, Bauer CE. 2003. Signal transduction by the global regulator RegB is mediated by a redox-active cysteine. EMBO J 22:4699–4708. doi:10.1093/emboj/cdg461. 12970182 PMC212728

[7] Wu J, Cheng Z, Reddie K, Carroll K, Hammad LA, Karty JA, Bauer CE. 2013. RegB kinase activity is repressed by oxidative formation of cysteine sulfenic acid. J Biol Chem 288:4755–4762. doi:10.1074/jbc.M112.413492. 23306201 PMC3576080

[8] Swem LR, Gong X, Yu CA, Bauer CE. 2006. Identification of a ubiquinone-binding site that affects autophosphorylation of the sensor kinase RegB. J Biol Chem 281:6768–6775. doi:10.1074/jbc.M509687200. 16407278 PMC2776112

[9] Wu J, Bauer CE. 2010. RegB kinase activity is controlled in part by monitoring the ratio of oxidized to reduced ubiquinones in the ubiquinone pool. mBio 1:e00272-10. doi:10.1128/mBio.00272-10. 21157513 PMC3000548

[10] Zhou A, Chen YI, Zane GM, He Z, Hemme CL, Joachimiak MP, Baumohl JK, He Q, Fields MW, Arkin AP, Wall JD, Hazen TC, Zhou J. 2012. Functional characterization of Crp/Fnr-type global transcriptional regulators in *Desulfovibrio vulgaris* Hildenborough. Appl Environ Microbiol 78:1168–1177. doi:10.1128/AEM.05666-11. 22156435 PMC3273024

[11] Kiley PJ, Beinert H. 1998. Oxygen sensing by the global regulator, FNR: the role of the iron-sulfur cluster. FEMS Microbiol Rev 22:341–352. doi:10.1111/j.1574-6976.1998.tb00375.x. 9990723

[12] Peuser V, Remes B, Klug G. 2012. Role of the Irr protein in the regulation of iron metabolism in *Rhodobacter sphaeroides*. PLoS One 7:e42231. doi:10.1371/journal.pone.0042231. 22879920 PMC3413700

[13] Imam S, Noguera DR, Donohue TJ. 2014. Global analysis of photosynthesis transcriptional regulatory networks. PLoS Genet 10:e1004837. doi:10.1371/journal.pgen.1004837. 25503406 PMC4263372

[14] Ke N, Kumka JE, Fang M, Weaver B, Burstyn JN, Bauer CE. 2022. RedB, a member of the CRP/Fnr family, functions as a transcriptional redox brake. Microbiol Spectr 10:e02353-22. doi:10.1128/Spectrum02353-22. PMC9603854 36106751

[15] Kimura M, Ishihama A. 1996. Subunit assembly *in vivo* of *Escherichia coli* RNA polymerase: role of the amino-terminal assembly domain of alpha subunit. Genes Cells 1:517–528. doi:10.1046/j.1365-2443.1996.d01-258.x. 9078382

[16] Ebright RH, Busby S. 1995. The *Escherichia coli* RNA polymerase alpha subunit: structure and function. Curr Opin Genet Dev 5:197–203. doi:10.1016/0959-437X(95)80008-5. 7613089

[17] Igarashi K, Ishihama A. 1991. Bipartite functional map of the *Escherichia coli* RNA polymerase alpha subunit. Involvement of the C-terminal region in transcription activation by cAMP-CRP. Cell 65:1015–1022. doi:10.1016/0092-8674(91)90553-b. 1646077

[18] Igarashi K, Hanamura A, Makino K, Aiba H, Aiba H, Mizuno T, Nakata A, Ishihama A. 1991. Functional map of the alpha subunit of *Escherichia coli* RNA polymerase: two modes of transcription activation by positive factors. Proc Natl Acad Sci USA 88:8958–8962. doi:10.1073/pnas.88.20.8958. 1833768 PMC52630

[19] Zou C, Fujita N, Igarashi K, Ishihama A. 1992. Mapping the cAMP receptor protein contact site on the alpha subunit of *Escherichia coli* RNA polymerase. Mol Microbiol 6:2599–2605. doi:10.1111/j.1365-2958.1992.tb01437.x. 1333035

[20] Bae B, Chen J, Davis E, Leon K, Darst SA, Campbell EA. 2015. CarD uses a minor groove wedge mechanism to stabilize the RNA polymerase open promoter complex. Elife 4:e08505. doi:10.7554/eLife.08505. PMC4593161 26349034

[21] Garner AL, Weiss LA, Manzano AR, Galburt EA, Stallings CL. 2014. CarD integrates three functional modules to promote efficient transcription, antibiotic tolerance, and pathogenesis in mycobacteria. Mol Microbiol 93:682–697. doi:10.1111/mmi.12681. 24962732 PMC4127138

[22] Weiss LA, Harrison PG, Nickels BE, Glickman MS, Campbell EA, Darst SA, Stallings CL. 2012. Interaction of CarD with RNA polymerase mediates *Mycobacterium tuberculosis* viability, rifampin resistance, and pathogenesis. J Bacteriol 194:5621–5631. doi:10.1128/JB.00879-12. 22904282 PMC3458692

[23] Srivastava DB, Leon K, Osmundson J, Garner AL, Weiss LA, Westblade LF, Glickman MS, Landick R, Darst SA, Stallings CL, Campbell EA. 2013. Structure and function of CarD, an essential mycobacterial transcription factor. Proc Natl Acad Sci USA 110:12619–12624. doi:10.1073/pnas.1308270110. 23858468 PMC3732983

[24] Valentini M, Storelli N, Lapouge K. 2011. Identification of C_4_-dicarboxylate transport systems in *Pseudomonas aeruginosa* PAO1. J Bacteriol 193:4307–4316. doi:10.1128/JB.05074-11. 21725012 PMC3165536

[25] Yamamoto H, Fang MX, Dragnea V, Bauer CE. 2018. Differing isoforms of the cobalamin binding photoreceptor AerR oppositely regulate photosystem expression. Elife 7:e39028. doi:10.7554/eLife.39028. 30281022 PMC6199135

[26] Fang MX, Bauer CE. 2017. The vitamin B_12_-dependent photoreceptor AerR relieves photosystem gene repression by extending the interaction of CrtJ with photosystem promoters. mBio 8:e00261-17. doi:10.1128/mBio.00261-17. 28325764 PMC5362033

[27] Cheng Z, Li KR, Hammad LA, Karty JA, Bauer CE. 2014. Vitamin B_12_ regulates photosystem gene expression via the CrtJ antirepressor AerR in *Rhodobacter capsulatus*. Mol Microbiol 91:649–664. doi:10.1111/mmi.12491. 24329562 PMC3946051

[28] Drews G. 2013. The intracytoplasmic membranes of purple bacteria—assembly of energy-transducing complexes. J Mol Microbiol Biotechnol 23:35–47. doi:10.1159/000346518. 23615194

[29] Elsen S, Colbeau A, Chabert J, Vignais PM. 1996. The *hupTUV* operon is involved in negative control of hydrogenase synthesis in *Rhodobacter capsulatus*. J Bacteriol 178:5174–5181. doi:10.1128/jb.178.17.5174-5181.1996. 8752335 PMC178314

[30] Dischert W, Vignais PM, Colbeau A. 1999. The synthesis of *Rhodobacter capsulatus* HupSL hydrogenase is regulated by the two-component HupT/HupR system. Mol Microbiol 34:995–1006. doi:10.1046/j.1365-2958.1999.01660.x. 10594824

[31] Jackson JB. 1995. Proton-translocating transhydrogenase and NADH dehydrogenase in anoxygenic photosynthetic bacteria, p 831–845. *In* Blankenship RE, Madigan MT, Bauer CE (ed), Anoxygenic photosynthetic bacteria. Advances in photosynthesis and respiration, vol 2. Kluwer Academic Publishers, Dordrecht, The Netherlands.

[32] Gray KA, Daldal F. 1995. Mutational studies of the cytochrome *bc*_1_ complexes, p 747–774. *In* Blankenship RE, Madigan MT, Bauer CE (ed), Anoxygenic photosynthetic bacteria. Advances in photosynthesis and respiration, vol 2. Kluwer Academic Publishers, Dordrecht, The Netherlands.

[33] Cecchini G. 2003. Function and structure of complex II of the respiratory chain. Annu Rev Biochem 72:77–109. doi:10.1146/annurev.biochem.72.121801.161700. 14527321

[34] McCrindle SL, Kappler U, McEwan AG. 2005. Microbial dimethylsulfoxide and trimethylamine-N-oxide respiration. Adv Microb Physiol 50:147–198. doi:10.1016/S0065-2911(05)50004-3. 16221580

[35] Mouncey NJ, Kaplan S. 1998. Redox-dependent gene regulation in *Rhodobacter sphaeroides* 2.4.1^T^: effects on dimethyl sulfoxide reductase (*dor*) gene expression. J Bacteriol 180:5612–5618. doi:10.1128/JB.180.21.5612-5618.1998. 9791109 PMC107618

[36] Mouncey NJ, Choudhary M, Kaplan S. 1997. Characterization of genes encoding dimethyl sulfoxide reductase of *Rhodobacter sphaeroides* 2.4.1^T^: an essential metabolic gene function encoded on chromosome II. J Bacteriol 179:7617–7624. doi:10.1128/jb.179.24.7617-7624.1997. 9401017 PMC179721

[37] Borghese R, Turina P, Lambertini L, Melandri BA. 1998. The *atpIBEXF* operon coding for the F-0 sector of the ATP synthase from the purple nonsulfur photosynthetic bacterium *Rhodobacter capsulatus*. Arch Microbiol 170:385–388. doi:10.1007/s002030050657. 9818357

[38] Laguna R, Joshi GS, Dangel AW, Luther AK, Tabita FR. 2010. Integrative control of carbon, nitrogen, hydrogen, and sulfur metabolism: the central role of the Calvin-Benson-Bassham cycle. Adv Exp Med Biol 675:265–271. doi:10.1007/978-1-4419-1528-3_15. 20532746

[39] Cukras AR, Green R. 2005. Multiple effects of S13 in modulating the strength of intersubunit interactions in the ribosome during translation. J Mol Biol 349:47–59. doi:10.1016/j.jmb.2005.03.075. 15876367 PMC1687178

[40] Studer SM, Joseph S. 2006. Unfolding of mRNA secondary structure by the bacterial translation initiation complex. Mol Cell 22:105–115. doi:10.1016/j.molcel.2006.02.014. 16600874

[41] Hauryliuk V, Ehrenberg M. 2006. Two-step selection of mRNAs in initiation of protein synthesis. Mol Cell 22:155–156. doi:10.1016/j.molcel.2006.04.004. 16630885

[42] Tedin K, Resch A, Blasi U. 1997. Requirements for ribosomal protein S1 for translation initiation of mRNAs with and without a 5′ leader sequence. Mol Microbiol 25:189–199. doi:10.1046/j.1365-2958.1997.4421810.x. 11902720

[43] Sorensen MA, Fricke J, Pedersen S. 1998. Ribosomal protein S1 is required for translation of most, if not all, natural mRNAs in *Escherichia coli in vivo*. J Mol Biol 280:561–569. doi:10.1006/jmbi.1998.1909. 9677288

[44] Pletnev P, Guseva E, Zanina A, Evfratov S, Dzama M, Treshin V, Pogorel’skaya A, Osterman I, Golovina A, Rubtsova M, Serebryakova M, Pobeguts OV, Govorun VM, Bogdanov AA, Dontsova OA, Sergiev PV. 2020. Comprehensive functional analysis of Escherichia coli ribosomal RNA methyltransferases. Front Genet 11:97. doi:10.3389/fgene.2020.00097. 32174967 PMC7056703

[45] Lakshmipathy SK, Gupta R, Pinkert S, Etchells SA, Hartl FU. 2010. Versatility of trigger factor interactions with ribosome-nascent chain complexes. J Biol Chem 285:27911–27923. doi:10.1074/jbc.M110.134163. 20595383 PMC2934658

[46] Deuerling E, Schulze-Specking A, Tomoyasu T, Mogk A, Bukau B. 1999. Trigger factor and DnaK cooperate in folding of newly synthesized proteins. Nature 400:693–696. doi:10.1038/23301. 10458167

[47] Teter SA, Houry WA, Ang D, Tradler T, Rockabrand D, Fischer G, Blum P, Georgopoulos C, Hartl FU. 1999. Polypeptide flux through bacterial Hsp70: DnaK cooperates with trigger factor in chaperoning nascent chains. Cell 97:755–765. doi:10.1016/S0092-8674(00)80787-4. 10380927

[48] Agashe VR, Guha S, Chang HC, Genevaux P, Hayer-Hartl M, Stemp M, Georgopoulos C, Hartl FU, Barral JM. 2004. Function of trigger factor and DnaK in multidomain protein folding: increase in yield at the expense of folding speed. Cell 117:199–209. doi:10.1016/S0092-8674(04)00299-5. 15084258

[49] Chan KK, Wood BM, Fedorov AA, Fedorov EV, Imker HJ, Amyes TL, Richard JP, Almo SC, Gerlt JA. 2009. Mechanism of the orotidine 5′-monophosphate decarboxylase-catalyzed reaction: evidence for substrate destabilization. Biochemistry 48:5518–5531. doi:10.1021/bi900623r. 19435314 PMC2697262

[50] Fleischhacker AS, Kiley PJ. 2011. Iron-containing transcription factors and their roles as sensors. Curr Opin Chem Biol 15:335–341. doi:10.1016/j.cbpa.2011.01.006. 21292540 PMC3074041

[51] Khoroshilova N, Popescu C, Munck E, Beinert H, Kiley PJ. 1997. Iron-sulfur cluster disassembly in the FNR protein of *Escherichia coli* by O_2_: [4Fe-4S] to [2Fe-2S] conversion with loss of biological activity. Proc Natl Acad Sci USA 94:6087–6092. doi:10.1073/pnas.94.12.6087. 9177174 PMC21006

